# An Uncommon Case of Ectopic Adrenocorticotropic Hormone Syndrome from a Pancreatic Neuroendocrine Tumor

**DOI:** 10.7759/cureus.4076

**Published:** 2019-02-14

**Authors:** Wen Wang, Ruoyu Miao, Ling Zhang, Syed Askari Hasan, Parkash Bakhtiani

**Affiliations:** 1 Internal Medicine, Florida Hospital, Orlando, USA

**Keywords:** ectopic acth syndrome, neuroendocrine tumor, pancreatic tumor, cushing syndrome, acth dependent cushing syndrome

## Abstract

Ectopic adrenocorticotropic hormone (ACTH) syndrome is a rare form of Cushing disease (CD) with over-secretion of ACTH from nonpituitary tumor outside the adrenal or pituitary glands. Its diagnosis relies on both biochemical tests (high-dose dexamethasone suppression test, ACTH level, corticotropin-releasing hormone test) to confirm ACTH-dependent CD and image studies (CT or MRI of chest, abdomen, and/or pelvis) for source localization. We present a rare case of ectopic ACTH syndrome from a pancreatic neuroendocrine tumor (NET).

## Introduction

Ectopic adrenocorticotropic hormone (ACTH) syndrome (EAS) is one type of Cushing disease (CD) where the source of ACTH is outside the pituitary gland. It is associated with a variety of benign or malignant tumor types, predominantly pulmonary, thymic, and pancreatic carcinoids, small cell lung carcinoma, pheochromocytoma, medullary thyroid carcinoma, and prostate carcinoma [1-5]. EAS rarely can be associated with nonendocrine, nonpulmonary tumors. Here we report a rare case of ectopic ACTH syndrome from a pancreatic neuroendocrine tumor (NET).

## Case presentation

A sixty-four-year-old female with a past medical history of newly diagnosed hypertension (HTN), type two diabetes mellitus, and hyperlipidemia presented with worsening generalized weakness and bilateral lower extremity swelling for two to three months. She also reported occasional flushing and easy bruising. She gained 20 pounds over six months with centrally distributed fat.

She was recently diagnosed with HTN and diabetes type two by her primary care physician. Routine lab work also showed hypokalemia. Examination revealed high blood pressure and tachycardia with two plus pretibial pitting edema bilaterally.

**Figure 1:**
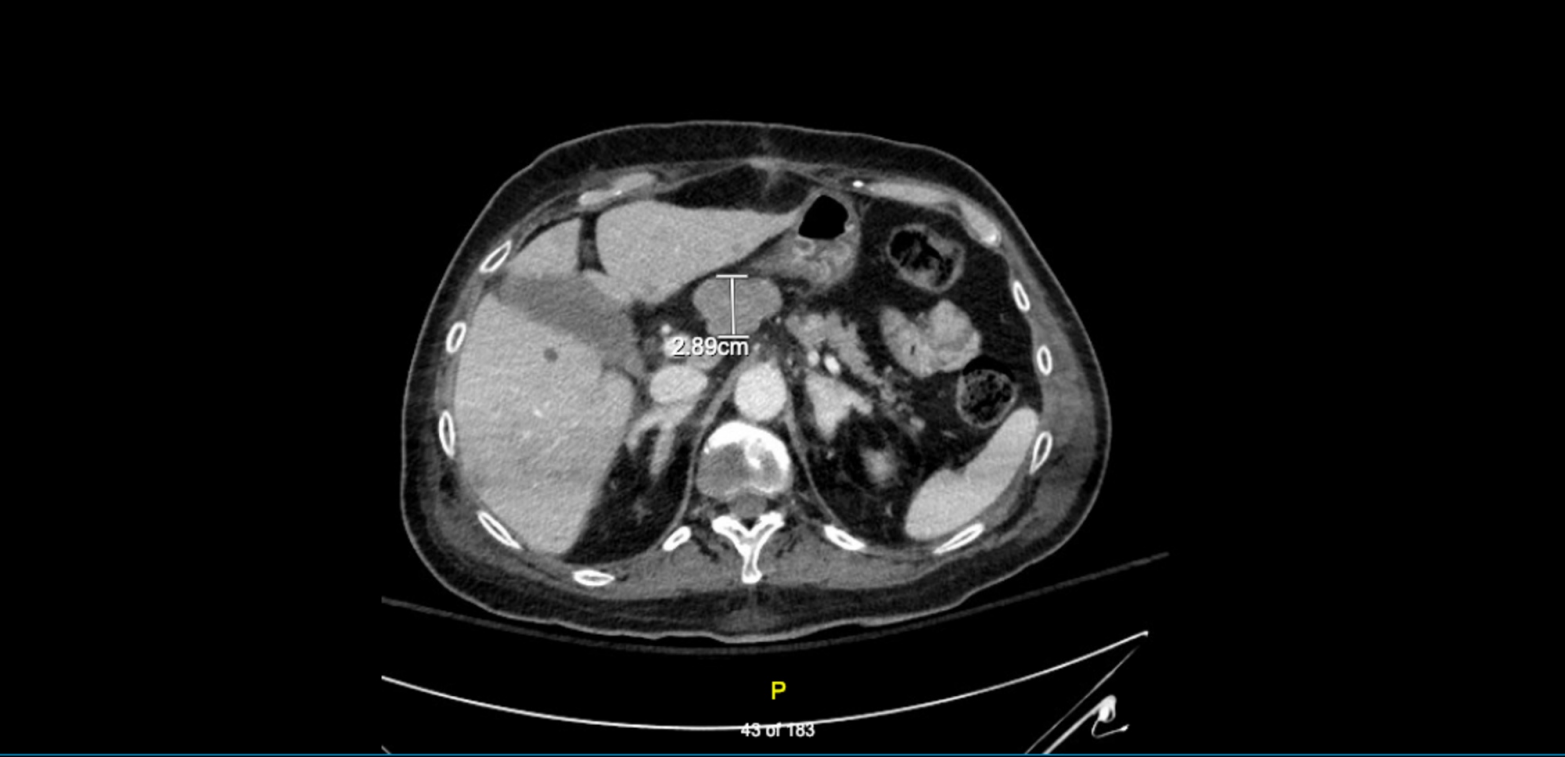
CT scan of abdomen with intravenous contrast. There is an ill-defined hypo-enhancing mass in the superior aspect of the pancreatic head which measures approximately 3.2 cm x 2.9 cm.

Due to the combination of recently diagnosed HTN and diabetes type two with hypokalemia, the patient underwent a workup for Cushing syndrome. Cortisol level in the early morning was 45 ug/dL (normal range: 7–28 μg/dL), and ACTH was 444 pg/mL (normal range: 10––60 pg/mL). High-dose dexamethasone suppression test was positive. Prolactin, insulin-like growth factor 1 (IGF-1), and thyroid-stimulating hormone (TSH) were all within normal limits. Subsequently, the patient had an MRI of brain which was negative for any mass. However, the CT scan of abdomen/pelvis with contrast revealed a mass in the pancreas with multiple liver lesions and lymphadenopathy (Figure 1). Pathology of the pancreatic mass which was obtained via endoscopic ultrasound (EUS) confirmed a well-differentiated intermediate-grade NET. Subsequently tumor markers were checked. Carbohydrate antigen (CA) 19-9 was 41.1 U/mL (normal range: 0–35 U/mL). Carcinoembryonic antigen (CEA) was 4.7 ng/mL (normal range: 0–5 ng/mL). Chromogranin A was 210 ng/mL (normal range: 0–95 ng/mL). The patient was evaluated for possible Whipple procedure. However, she was deemed not a candidate due to significant liver tumor burden. Her clinical course deteriorated rapidly despite medical management. She passed away two months after the diagnosis of the pancreatic NET. 

## Discussion

Ectopic adrenocorticotropic hormone syndrome is rare and accounts for around 5%-10% of all types of CD [1-3, 5-8]. In the past, small cell lung carcinoma (3.3%-50%) represented the most frequent cause of EAS [3, 9]. However, with improved imaging techniques and changing referral patterns, other NETs (mainly bronchial carcinoids) have become the most common tumors that are associated with EAS in more recent studies [1-3, 5, 9]. Occult tumors comprise around 12%-38% of all EASs [5, 9]. It is even rarer to have pancreatic NET, which is reported to represent only about 7.5%-25% of EASs [9], and the incidence is lower than 0.1 per one million people [10]. Only a few cases have been reported so far. It is a very aggressive tumor; metastasis could occur in as high as 76%-88% of patients [9, 11], and the five-year survival rate could be as low as 16% [11].

The diagnosis of EAS is based on the combination of biochemical tests [high-dose dexamethasone suppression test, corticotrophin-releasing hormone (CRH), desmopressin stimulation test, and/or bilateral inferior petrosal sinus sampling (BIPSS)], measures of tumor markers, and imaging (pituitary MRI, thoraco-abdomino-pelvic CT, somatostatin receptor scintigraphy). The first step of diagnosis is to confirm CD. The second step of diagnosis is to distinguish ACTH-dependent CD [values greater than 15-20 pg/mL (3.3-4.4 pmol/L)] from ACTH-independent CD. In case of doubt, the CRH test or high-dose dexamethasone suppression test may be performed. The third step is to localize the ACTH-secreting tumor by imaging. BIPSS is the gold standard test to differentiate pituitary versus ectopic ACTH secretion, and the sensitivity and specificity are approximately 95% [3, 6]. Contrast-enhanced CT, MRI, and scintigraphic studies can localize ACTH-secreting tumors in 70%-90% of cases [1-3]. Considering the potentially high metastasis rate, complete staging with multimodality approach is, therefore, critical prior to treatment [3].

Ectopic adrenocorticotropic hormone syndrome may cause significant comorbidity in patients with underlying malignancy and severe hypercortisolism [3, 8]. Surgical removal of the tumor can result in full recovery in some cases; however, it may recur later on. If surgery is not an option due to severe metastatic disease, a multidisciplinary approach should be adopted to control tumor growth and associated symptoms. These approaches may include somatostatin analog therapy, peptide receptor radionuclide therapy, chemotherapy, radiotherapy, and/or targeted therapies [3]. In such cases, control of the hypercortisolemia can be achieved with long-term adrenolytic medication, such as methyrapone, ketoconazole, mitotane, or even etomidate [8, 12].

## Conclusions

Pancreatic NET is a rare cause of ACTH-dependent Cushing syndrome. Suspicion for this condition should be raised in patients without primary pituitary lesion and other apparent sources. Diagnosis is based on biochemical tests to confirm EAS and imaging studies for tumor localization. Treatment should focus on resection, chemo-radiation, and hormone analogs. Close follow-up is very important in this rare yet aggressive tumor.
